# Remodeling Functional Connectivity in Multiple Sclerosis: A Challenging Therapeutic Approach

**DOI:** 10.3389/fnins.2017.00710

**Published:** 2017-12-13

**Authors:** Mario Stampanoni Bassi, Luana Gilio, Fabio Buttari, Pierpaolo Maffei, Girolama A. Marfia, Domenico A. Restivo, Diego Centonze, Ennio Iezzi

**Affiliations:** ^1^Unit of Neurology & Unit of Neurorehabilitation, IRCCS Istituto Neurologico Mediterraneo Neuromed, Pozzilli, Italy; ^2^Multiple Sclerosis Research Unit, Department of Systems Medicine, Tor Vergata University, Rome, Italy; ^3^Department of Neurology, Nuovo Garibaldi Hospital, Catania, Italy

**Keywords:** multiple sclerosis, inflammation, brain networks, functional connectivity, synaptic plasticity, non-invasive brain stimulation

## Abstract

Neurons in the central nervous system are organized in functional units interconnected to form complex networks. Acute and chronic brain damage disrupts brain connectivity producing neurological signs and/or symptoms. In several neurological diseases, particularly in Multiple Sclerosis (MS), structural imaging studies cannot always demonstrate a clear association between lesion site and clinical disability, originating the “clinico-radiological paradox.” The discrepancy between structural damage and disability can be explained by a complex network perspective. Both brain networks architecture and synaptic plasticity may play important roles in modulating brain networks efficiency after brain damage. In particular, long-term potentiation (LTP) may occur in surviving neurons to compensate network disconnection. In MS, inflammatory cytokines dramatically interfere with synaptic transmission and plasticity. Importantly, in addition to acute and chronic structural damage, inflammation could contribute to reduce brain networks efficiency in MS leading to worse clinical recovery after a relapse and worse disease progression. These evidence suggest that removing inflammation should represent the main therapeutic target in MS; moreover, as synaptic plasticity is particularly altered by inflammation, specific strategies aimed at promoting LTP mechanisms could be effective for enhancing clinical recovery. Modulation of plasticity with different non-invasive brain stimulation (NIBS) techniques has been used to promote recovery of MS symptoms. Better knowledge of features inducing brain disconnection in MS is crucial to design specific strategies to promote recovery and use NIBS with an increasingly tailored approach.

## Introduction

Multiple Sclerosis (MS) is an autoimmune inflammatory disease of the central nervous system (CNS) characterized by white matter demyelinating lesions and gray matter atrophy. MS represents one of the most frequent neurological condition associated with clinical disability in young adults. Symptoms include a huge range of manifestations such as motor/sensory deficits, fatigue, spasticity, cognitive dysfunction, and pain, related to the different neural systems involved. In several neurological disorders, and particularly in MS, structural imaging studies hardly demonstrate clear associations between lesion site and clinical disability. The peculiar discordance between radiological and clinical features is usually referred to as the “clinico-radiological paradox” (Barkhof, 2002). The remote effects of a brain lesion on functionally connected regions and the ongoing rearrangement produced by synaptic plasticity in response to brain damage can concur in determining the discrepancy between structural damage and clinical symptoms.

The concept of diaschisis (von Monakow, 1914; Feeney and Baron, 1986) refers to focal changes in metabolism or neuronal activity in anatomically intact brain regions located away from the lesion (Carrera and Tononi, 2014). Recently, the concept of diaschisis has been applied to complex networks analysis. The connectome is defined as an overall map of neural connections in the brain (Sporns et al., 2005) represented by a set of nodes (i.e., graphs) joined by lines depicted between them (Bullmore and Bassett, 2011). It is possible to explore how network activity is changed by a lesion relying upon its topography within the network architecture (Honey and Sporns, 2008; Alstott et al., 2009; Joyce et al., 2013). Accordingly, the term “connectomal diaschisis” refers to changes in the structural and functional connectome, including disconnections between and reorganization of subgraphs, involving areas located away from the lesion (Carrera and Tononi, 2014). A better definition of diaschisis could contribute to clarify the clinico-radiological paradox in neurological disorders. Understanding how lesions alter brain networks could help to select the appropriate treatment based on the underlying process. However, it is challenging to ascertain whether remote connectivity changes occur following diaschisis or rely on other recovery mechanisms, such as plasticity (either positive or maladaptive) and vicariation (Carrera and Tononi, 2014).

Clinical improvement after brain lesions mainly depends on structural and functional connectivity restoring. Synaptic plasticity is the main mechanism involved, both promoting spontaneous recovery and mediating the beneficial effects of rehabilitation. Non-invasive brain stimulation (NIBS) techniques such as repetitive transcranial magnetic stimulation (rTMS) and transcranial direct current stimulation (tDCS) perturbing local neural activity can subsequently affect the function of distributed brain regions located away from the stimulated area. Therefore, NIBS can be successfully used for testing and modulating brain networks dynamics in physiological and in a number of neuropsychiatric conditions (Shafi et al., 2012).

A number of studies suggest that inflammatory molecules released during MS relapses alter neuronal functioning acting both on synaptic transmission and plasticity (Stampanoni Bassi et al., 2017b). It is therefore reasonable to assume that inflammation in MS could disrupt brain connectivity, even regardless of demyelinating white matter lesions and gray matter atrophy. In addition, as inflammation could restrain brain network reorganization inducing synaptic plasticity alterations, promoting beneficial synaptic plasticity through NIBS could represent a promising therapeutic approach in MS.

In this paper, the main studies exploring brain connectivity in MS with different techniques will be overviewed. We performed a literature search in PubMed in August 2017 using the terms “connectivity” and “multiple sclerosis.” We looked for original case-control studies, case series, or cohort studies. We also examined many of the references of the articles found. We excluded studies not available in English language, studies conducted in animals, studies published more than 10 years before, and studies including patients with age <18 years. We will also overview the alterations of synaptic transmission and plasticity described in a MS experimental model (i.e., experimental autoimmune encephalomyelitis, EAE) and in human MS using TMS. The findings supporting the role of inflammation in inducing connectivity dysfunction and the possible role of NIBS in promoting beneficial connectivity for recovery will be discussed.

## Connectivity in MS

**Figure 1:**
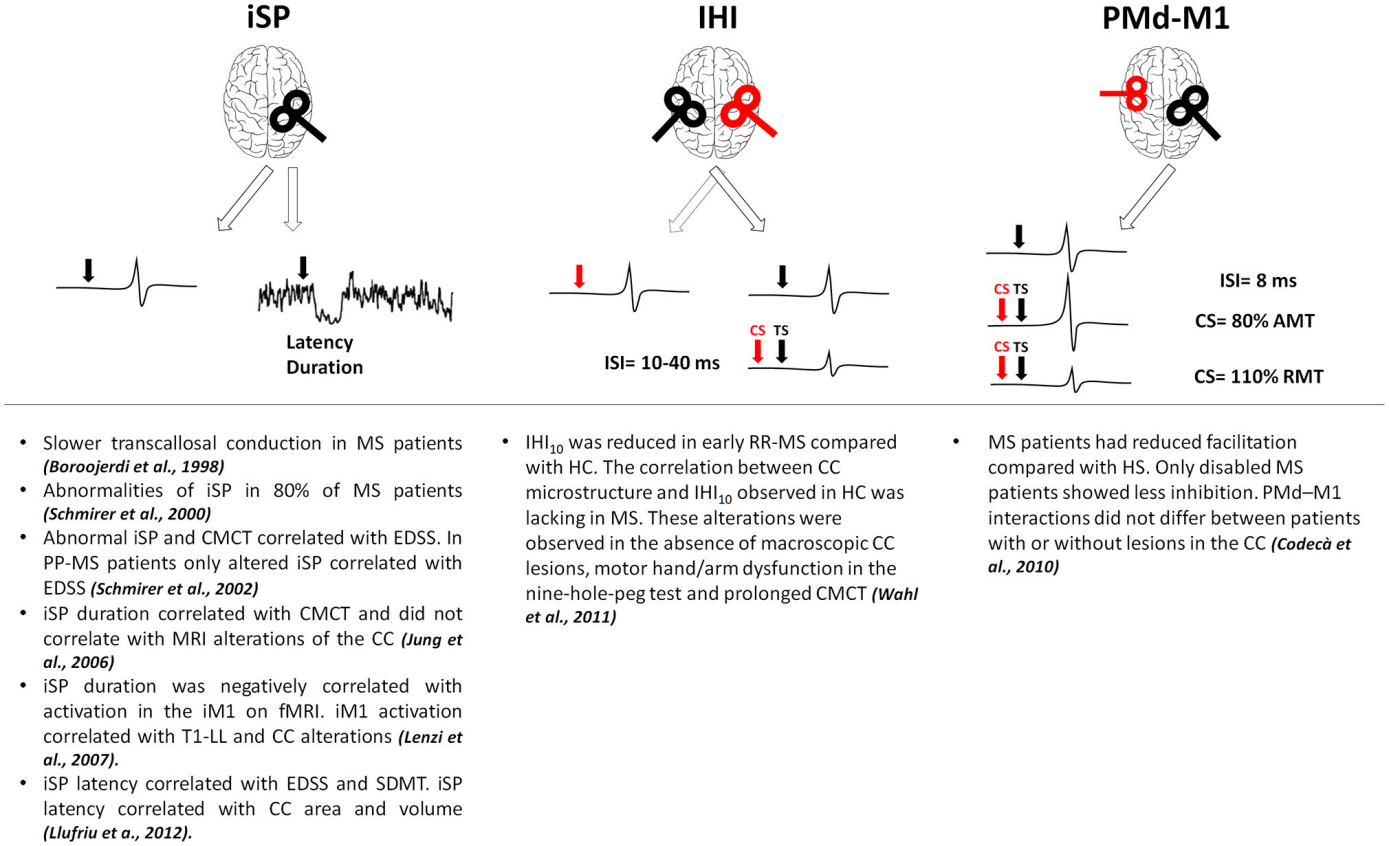
Results of the main studies investigating interhemispheric connections in MS. Red coils and arrows represent CS, black coils and arrows depict TS. AMT, active motor threshold; CC, corpus callosum; CMCT, central motor conduction time; CS, conditioning stimulus; EDSS, expanded disability status scale; fMRI, functional magnetic resonance imaging; HC, healthy controls; IHI, interhemispheric inhibition; iM1, ipsilateral primary motor cortex; ISI, interstimulus interval; iSP, ipsilateral silent period; M1, primary motor cortex; PMd, dorsal premotor cortex; PP-MS, primary progressive multiple sclerosis; RMT, resting motor threshold; RR-MS, relapsing-remitting multiple sclerosis; SDMT, symbol digit modality test; T1-LL, T1 lesion load; TS, test stimulus.

In MS, alterations of brain connectivity have been studied with different techniques. TMS has been used to test cortico-cortical connectivity, checking how a stimulus delivered on a given brain region can influence the excitability of a different region and providing information on effective connectivity (Gerstein and Aertsen, 1985; Friston et al., 1993). Inhibitory connectivity between homologous regions of both primary motor cortices (M1) has been assessed using either a single coil or a double-coil (d-c) approach (Figure 1). With a single suprathreshold magnetic pulse given over M1, it is possible to induce inhibitory influences in the contralateral M1 measurable as a suppression of the tonic muscle voluntary activity ipsilateral to the stimulated cortex (ipsilateral silent period, iSP; Wassermann et al., 1991). Interhemispheric inhibition (IHI) can be also studied with d-c TMS when a suprathreshold stimulus delivered over one M1 is able to suppress the test response elicited by a suprathreshold stimulus given over the contralateral M1 at short (10 ms) or longer (40 ms) interstimulus intervals (Ferbert et al., 1992; Murase et al., 2004; Uehara et al., 2013). The two subtypes of IHI are likely mediated by different physiological mechanisms, both depending on GABAB transmission (Kukaswadia et al., 2005; Radhu et al., 2012). IHI40 could depend on an overlapping population of inhibitory neurons activated by the excitatory input from the contralateral M1 (Kukaswadia et al., 2005) whereas IHI10 may be mediated by transcallosal fibers passing through the posterior body and the isthmus of the corpus callosum (CC, Ni et al., 2009). In MS, owing to high prevalence of lesions within the CC, most TMS approaches mainly focused on interhemispheric connectivity (Boroojerdi et al., 1998; Schmierer et al., 2000; Codecà et al., 2010; Wahl et al., 2011). In MS, altered iSP correlated with clinical disability (Schmierer et al., 2000, 2002; Llufriu et al., 2012; Neva et al., 2016) and with central motor conduction time prolongation (Jung et al., 2006). Whereas, some studies showed a correlation between CC lesions and iSP alterations (Lenzi et al., 2007; Llufriu et al., 2012), other studies found that reduced iSP did not correlate with magnetic resonance imaging (MRI) alterations of the CC (Jung et al., 2006). One study reported reduced IHI in early RR-MS even without detectable CC lesions at conventional MRI, making IHI failure a possible marker of callosal disconnection also at earlier disease stages (Wahl et al., 2011). Finally, altered connectivity between dorsal premotor cortex and contralateral M1 suggests that also excitatory transcallosal connectivity may be impaired independently of lesion load and site, and even in the absence of clinical disability (Codecà et al., 2010).

Recently, functional MRI (fMRI) gave the opportunity to study the activity of a large number of brain regions. FMRI is a tool able to reveal dynamic changes in brain tissue occurring whilst the subject is awake and fully relaxed (i.e., resting state fMRI, rs-fMRI), or in response to specific behavioral tasks (i.e., task-based fMRI). In MS, task-based fMRI showed increased and widespread brain activations compared to healthy controls, particularly at disease onset (Rocca et al., 2005). Increased activation is generally interpreted as adaptive plastic changes aimed at preventing the clinical manifestations (Pantano et al., 2006; Filippi and Rocca, 2013). Nevertheless, overactivation does not consequently denote adaptive plasticity, as it may be associated to high disability. For instance, diffuse microstructural damage—as shown by combined diffusion tensor imaging (DTI) and fMRI- correlated with increased sensorimotor network activation (Rocca et al., 2002; Lenzi et al., 2007). Intriguingly, increased activation of ipsilateral M1 during hand movements correlated with CC damage and loss of transcallosal inhibition (Lenzi et al., 2007) suggesting that ipsilateral M1 hyperactivation could likely represent a simple epiphenomenon of disease.

The development of methods testing brain connectivity at rest helped to avoid behavioral confounding related to task. In particular, the temporal correlation between neural or hemodynamic spontaneous activity arising from distinct brain regions, namely functional connectivity (FC), describes the intrinsic property of a given area or the influences of a particular area over another region, independently of external stimuli (Biswal et al., 1995; Fox and Raichle, 2007; van den Heuvel and Hulshoff Pol, 2010). In brain networks analysis different types of connectivity can be explored. Structural connectivity is usually referred to the anatomical connections and is evaluated by fiber tractography from DTI to obtain a reliable map of anatomical connection between brain areas. The relationship between structural and functional brain networks has not been yet completely elucidated (Rubinov et al., 2009; Honey et al., 2010; Ponten et al., 2010). Whereas, areas anatomically connected show a greater FC (Honey et al., 2007, 2009; Rubinov et al., 2009; Hermundstad et al., 2013), functional interactions are not limited to directly connected areas (Honey et al., 2007, 2009). A better definition of the complex relationship between structural and functional brain networks could predict how structural damage alters network dynamics (Honey and Sporns, 2008; Kaiser, 2013; van Dellen et al., 2013).

In MS, several studies have described rs-fMRI alterations involving different networks. Increased or decreased FC have been related to disease phenotype, clinical characteristics and neuroradiological findings (Table 1). Overall, findings apparently contrast likely due to different methodological approaches, and different clinical phenotypes. In addition, whether FC changes could be compensatory or maladaptive is not yet clear. Longitudinal studies or combining different experimental techniques could contribute to clarify the clinical implications of connectivity changes in MS. In addition, standardized measures should be properly identified to predict the effect of damage, evaluate symptoms evolution and compare the effect of different treatment protocols. Recently, graph based rs-fMRI studies showed that analyzing properties of large-scale brain networks could help to identify reliable measures of brain network functioning. Parameters such as clustering coefficient, path length, network centrality, and modularity, show that brain networks magnify cost efficiency of parallel information processing (Achard and Bullmore, 2007). Moreover, these properties contribute to protect networks from damage (Achard et al., 2006; Achard and Bullmore, 2007). Accordingly, clinical symptoms may occur in MS when structural damage raises to a critical level reducing overall network efficiency (Schoonheim et al., 2015).

**Table 1:** Main results of studies investigating rs-FC in MS.

**Authors**	**Study population**	**Main findings**
Roosendaal et al., 2010	14 CIS, 31 RR-MS, 41 HCs	Increased rs-FC in several RSNs, including the DMN and sensorimotor network, in CIS compared to HCs and RR-MS. In RR-MS GM atrophy and abnormal WM diffusivity compared to HCs. No changes in CIS. rs-FC and structural MRI or clinical disability did not correlate in CIS and RR-MS.
Rocca et al., 2010	33 SP-MS (18 CI), 24 PP-MS (12 CI), 24 HCs	In SP-MS reduced activity in the DMN, in the medial prefrontal cortex and precentral gyrus, compared with HCs. In PP-MS reduced activity in the precentral gyrus and the ACC compared with HCs. In SP-MS increased ACC activity compared with PP-MS. RS activity in the ACC was reduced particularly in CI. DMN abnormalities correlated with cognitive test and DTI changes in the CC and the cingulum.
Bonavita et al., 2011	36 RR-MS (18 CI and 18 CP), 18 HCs	In RR-MS reduced DMN connectivity in the ACC, reduced in the core and increased at the periphery of the PCC. No correlations between FC changes and global atrophy or T2-LL, but association with regional GM loss. The findings were more marked in CP than CI.
Faivre et al., 2012	13 early RR-MS, 14 HCs	Increased rs-FC in several RSNs in early RR-MS compared with HCs. No correlations between RSNs connectivity and T2-LL or disease duration. Increased rs-FC in cognitive and sensorimotor networks negatively correlated with different cognitive and motor tasks and MSFC scores.
Rocca et al., 2012	85 RR-MS, 40 HCs	In RR-MS decreased rs-FC in different RSNs (salience, executive control, working memory, default mode, sensorimotor, and visual) and increased rs-FC in regions of the executive control and auditory RSNs. Decreased rs-FC was correlated with disability and T2 lesion volumes.
Tona et al., 2014	48 RR-MS, 24 HCs	In RR-MS both increased and decreased connectivity within the thalamic RSN. No significant correlation between thalamic FC and radiologic variables. Increased thalamocortical FC correlated with decreased cognitive performance.
Cruz-Gómez et al., 2014	60 RR-MS (30 CI and 30 CP), 18 HCs	Decreased rs-FC in the DMN in CI compared with CP and HCs. Decreased rs-FC in the LFPN in both CI and CP compared with HCs. Decreased rs-FC in the RFPN and salience network in CI compared with CP. BPF correlated with rs-FC in the DMN, LFPN and RFPN. T1-LL negatively correlated with rs-FC in all explored RSNs.
Louapre et al., 2014	35 RR-MS (15 CI and 20 CP), 20 HCs	In CI decreased rs-FC in DMN and ATT compared with CP. In CI decreased rs-FC particularly between the medial prefrontal cortex and the PCC, predicted by PCC atrophy. In CI higher WM LL and more severe GM atrophy in cognitive networks compared with CP. In CP increased rs-FC in ATT compared with HCs.
Zhou et al., 2014	24 RR-MS and 24 HC	The connections of paired DMN subregions showed decreased SC and increased FC in RR-MS patients. SC alterations correlated with EDSS. Decreased SC was correlated to atrophy.
Rocca et al., 2015	69 CP MS, 42 HCs	In CP MS decreased rs-FC between the hippocampi and several cortical–subcortical regions within the DMN. Reduced hippocampal rs-FC correlated with T2-LL, disease duration, depression and disability.
Sbardella et al., 2015	30 RR-MS and 24 HCs	In RR-MS decreased rs-FC in several networks (cerebellar, executive-control, medial-visual, basal ganglia and sensorimotor) and changes in inter-network correlations. CC microstructural damage correlated with FC in cerebellar and auditory networks. No correlations between rs-FC in all explored RSNs and T2-LL.
Liu et al., 2015	35 RR-MS, 35 HC	Compared to HC, the MS group showed significantly decreased FC between thalamus and several brain regions including right middle frontal and parahippocampal gyri, and the left inferior parietal lobule. Increased intra and inter-thalamic FC was observed in MS compared to HC. FC alterations were not correlated with T2-LL, thalamic volume or the presence of thalamic lesions.
Baltruschat et al., 2015	17 RR-MS, 15 HCs	In RR-MS increased FC between left posterior cingulate gyrus/precuneus, and left middle temporal gyrus and left cerebellum. In RR-MS GM bilateral atrophy in posterior cingulate gyrus/precuneus. BPF negatively correlated with FC between left posterior cingulate gyrus/precuneus and left cerebellum.
Rocca et al., 2016	214 MS patients (RR-MS and SP-MS), 55 HC	Global network properties (network degree, global efficiency, hierarchy, path length and assortativity) were abnormal in MS compared with HC and contributed to distinguish CI MS patients from HC, but not the main MS phenotypes. In MS, global and regional network properties were not correlated with T2-LL and normalized brain volume.
d'Ambrosio et al., 2017	187 MS patients (136 RR-MS, 42 SP-MS and 9 PP- MS), (122 CP and 65 CI); 94 HCs	In patients lower GM, WM and thalamic volumes compared with HCs. In patients decreased rs-FC between thalamic subregions and the caudate, cingulate cortex and cerebellum correlated with worse motor performance. Increased rs-FC with the insula correlated with better motor performance. In CI increased rs-FC between thalamic subregions and temporal areas compared with CP. No correlations between thalamic rs-FC and T2-LL.

*ACC, anterior cingulate cortex; ATT, attentional network; CC, corpus callosum; CI, cognitive-impaired patients; CIS, clinically isolated syndrome; CP, cognitive-preserved patients; DMN, Default Mode Network; DTI, Diffusion Tensor Imaging; FC, functional connectivity; GM, gray matter; HCs, healthy controls; LFPN, left fronto-parietal network; LL, lesion load; MRI, Magnetic Resonance Imaging; MS, Multiple Sclerosis; MSFC, Multiple Sclerosis Functional Composite Score; PCC, posterior cingulate cortex; PP-MS, primary-progressive MS patients; RFPN, right fronto-parietal network; RR-MS, relapsing-remitting MS patients; rs-FC, resting-state Functional Connectivity; RSN, resting state network; SC, structural connectivity; SP-MS, secondary-progressive MS patients; WM, white matter*.

## Inflammation alters synaptic transmission and brain connectivity

In MS, brain connectivity disruption could rely on the acute and chronic structural damage and also on inflammation. In MS experimental models, inflammatory cytokines induce alterations of synaptic transmission of both glutamatergic and GABAergic transmission, causing synaptic hyperexcitability (Rossi et al., 2011; Mandolesi et al., 2013; Stampanoni Bassi et al., 2017a). Interleukin (IL)-1β represents one of the main inflammatory cytokines involved (Mandolesi et al., 2013).

Synaptic transmission can be explored in humans with TMS and specific protocols are related to different features of synaptic transmission (Ziemann et al., 2008; Rossini et al., 2015). Studies in MS showed that the same inflammatory cytokines induce synaptic alterations similar to those seen in EAE (Rossi et al., 2012a); moreover, the magnitude of these alterations correlated with CNS levels of IL-1β. Furthermore, cerebrospinal fluid from MS patients in active phase of the disease reproduced *in vitro* both the glutamatergic and GABAergic alterations and the neuronal degeneration observed in EAE (Rossi et al., 2012a,b). In addition, different phases and disease phenotypes are associated to specific patterns of alterations. In particular, the relapsing phase of MS is characterized by cortical disinhibition as indexed by reduced contralateral silent period duration and reduced short-interval intracortical inhibition (Caramia et al., 2004).

As inflammatory cytokines alter synaptic functioning, a direct role of neuroinflammation in connectivity dysfunction occurring in MS may be hypothesized. To support this view, few studies showed that in patients with clinically isolated syndrome (CIS) significant FC alterations develop even without white matter lesions or brain atrophy. One study showed significant rs-fMRI changes in a group of patients with CIS manifesting as acute optic neuritis, involving both left and right primary visual cortices and extravisual regions (Wu et al., 2015). Another study explored FC alterations in a group of RR-MS patients and in a group of patients with CIS without brain lesions (Liu et al., 2016), including patients with optic neuritis or spinal cord syndromes. CIS patients showed significantly decreased FC in the visual areas and increased FC in the temporal lobes. It should be highlighted that in both studies altered FC developed also in networks other than the visual system, likely suggesting that acute inflammation may induce diffuse connectivity changes.

It is important to mention another study exploring FC in CIS and RR-MS patients considering graph-based network analysis (Liu et al., 2017). As expected, in RR-MS patients decreased whole brain network efficiency, reduced nodal efficiency, and impaired FC were found. In addition, patients with CIS displayed a similar pattern of alterations. In particular, impaired FC involved the occipital, temporal, and frontal cortices and the insula. Finally, changes in RR-MS did not correlate with white matter lesion load and site, and with gray matter atrophy.

A number of studies showed that also systemic inflammation could affect brain FC. For instance, in healthy individuals, experimental inflammation influences brain activity in the insula and in the cingulate cortex (Hannestad et al., 2012) and alters resting connectivity between the left thalamus and the right posterior cingulate cortex (Labrenz et al., 2016). Furthermore, a study using graph analysis showed that in patients with Hepatitis C the administration of IFN α reduced whole brain network connectivity and efficiency (Dipasquale et al., 2016). Recently, a study showed that IL-6 blood levels covaried with connectivity in the default mode network (Marsland et al., 2017). These studies are in line with previous reports showing that peripheral cytokines may modulate central synaptic transmission altering task-based fMRI (Capuron et al., 2005; Harrison et al., 2009).

Overall, these data suggest that inflammation in MS, altering synaptic transmission, may represent an additional key feature contributing to network dysfunction. It may be hypothesized that, in addition to structural damage, network efficiency can be dramatically disrupted by inflammatory cytokines.

## Inflammation, synaptic plasticity, and clinical recovery

Recovery after brain injury mainly depends on the ability of surviving neurons to undergo long-term functional changes (Floel and Cohen, 2006). Long-term potentiation (LTP), the most studied form of synaptic plasticity, consists of enduring enhancement of synaptic strength followed by structural rearrangements (Bliss and Gardner-Medwin, 1973). LTP can be virtually induced in all brain areas and may reduce the clinical expression of neuronal damage likely restoring the excitability of neurons deprived of their synaptic inputs. Promoting LTP could therefore contribute to maximize network efficiency restoring, delaying the clinical expression of brain damage. The link between LTP and clinical recovery after acute brain lesion first came from animal studies. In rats, neurological deficit after experimental ischemia partly improved 7 days after the infarction and correlated with increased glutamatergic excitatory transmission in surviving neurons, suggesting that recovery was driven by increased excitatory synaptic activity surrounding the damaged area (Centonze et al., 2007c). In humans, the evidence that LTP could be crucial for clinical recovery was first showed in acute stroke patients, comparing the amount of TMS-induced LTP-like plasticity with the degree of recovery 6 months later. Patients with higher TMS-induced LTP displayed a better recovery (Di Lazzaro et al., 2010a). Accordingly, the term LTP reserve has been proposed to indicate the amount of LTP induced by different TMS protocols as significant predictor of clinical recovery.

Physical rehabilitation represents the main treatment option to enhance spontaneous recovery of neurological deficits Albeit early rehabilitation after acute brain lesions can facilitate recovery, the optimal treatment type is still poorly defined (Morreale et al., 2016). It is likely that the beneficial effect of rehabilitation could be mediated by LTP, as shown in animals and also in human studies by the findings that learning a motor skill engages LTP mechanisms triggered by motor practice (Rioult-Pedotti et al., 2000; Muellbacher et al., 2001; Ziemann et al., 2004).

Different studies reported alterations of synaptic plasticity in EAE and MS (Mori et al., 2011, 2012; Di Filippo et al., 2013; Nisticò et al., 2013). An early finding was that MS relapses are associated with impaired LTP-like plasticity as assessed with intermittent theta-burst stimulation (iTBS) (Mori et al., 2011). In a further study, iTBS-induced LTP-like plasticity was explored in RR-MS patients either in acute or stable disease phase, confirming that LTP-like plasticity is reduced in relapsing patients compared to remitting patients (Mori et al., 2012) Remarkably, in the same study, 6-months treatment with interferon (IFN) beta 1-a improved LTP-like plasticity in relapsing patients (Mori et al., 2012).

The association between LTP reserve and clinical recovery has been explored in RR-MS patients using the paired associative stimulation protocol (Mori et al., 2014). In this study, LTP reserve tested at the time of a clinical relapse correlated with clinical recovery 3 months later. Patients with greater LTP reserve showed a better recovery whereas patients with absent or poor LTP reserve displayed partial or absent clinical recovery, further suggesting the crucial role of LTP taking place in surviving neurons to compensate coexisting neuronal loss (Mori et al., 2014). Overall, these studies suggest that CNS inflammation in MS patients negatively influences pathways involved in LTP induction and maintenance (Tongiorgi et al., 2012; Stampanoni Bassi et al., 2017a). In addition, some studies suggest that negative impact of acute inflammation on LTP may be reduced by immune-modulating therapies (Mori et al., 2012; Di Filippo et al., 2016). In MS, it is likely that inflammation-induced LTP alterations could lessen brain network reorganization influencing clinical recovery after a relapse and disease progression and that resolving inflammation could positively influence clinical recovery.

## NIBS and symptoms recovery in MS

NIBS has been used in healthy subjects for enhancing motor skills and cognitive functions, and in neurological and psychiatric patients for therapeutic purposes (Hummel et al., 2005; Miniussi et al., 2008). Different studies showed that focal perturbation of neural activity by NIBS selectively modulates functional and effective connectivity in different connected networks (Grefkes et al., 2010; Eldaief et al., 2011; Grefkes and Fink, 2011; Cocchi et al., 2016). Furthermore, as inflammation alters synaptic plasticity, boosting LTP through NIBS could help to improve recovery in MS patients.

rTMS and tDCS represent the most commonly used NIBS techniques able to induce LTP-like plasticity (Ziemann et al., 2008). In M1, this plastic effect can be generally measured as an increase of the peak-to-peak amplitude of the motor evoked potentials (MEPs) after the plasticity-inducing protocol, likely coming from enhanced excitability of the cortico-cortical facilitatory synaptic inputs onto the corticospinal cells (Di Lazzaro et al., 2010b). A number of rTMS protocols are able to induce persistent changes in cortical excitability depending on the intensity, frequency and number of stimuli applied, frequency playing a pivotal role. In particular, high-frequency (i.e., ≥5 Hz) rTMS protocols produce LTP-like plasticity (Siebner and Rothwell, 2003; Ziemann et al., 2008). Subsequently, new rTMS protocols have been introduced to modulate cortical plasticity, including TBS. In particular, iTBS produces excitatory after-effects through LTP-like plasticity (Huang et al., 2005). tDCS employs weak transcranial currents to induce changes in cortical excitability depending on stimulation polarity (Nitsche and Paulus, 2000). In particular, anodal tDCS may entail LTP-like mechanisms (Liebetanz et al., 2002; Nitsche et al., 2003).

NIBS techniques have been applied to treat different symptoms in MS patients. Spasticity is considered as the consequence of the hyperexcitability of the stretch reflex secondary to corticospinal tract lesions and reduced supraspinal inhibitory input (Young, 1994). In MS, a single session of 5 Hz rTMS over M1 reduced stretch reflex hyperexcitability (Centonze et al., 2007a). Moreover, daily application for 2 weeks of both 5 Hz rTMS and iTBS can be useful to reduce lower limb spasticity in MS (Centonze et al., 2007a; Mori et al., 2010a). Noticeably, one longitudinal study showed that repeated daily iTBS sessions associated with physical rehabilitation may induce functional reorganization of M1 connectivity. In particular, reduced spasticity was associated with both changes in local connectivity and in the inter-hemispheric balance (Boutière et al., 2017). Conversely, one study reported that anodal tDCS on M1 for 5 consecutive days had no clinical impact on spasticity in MS patients (Iodice et al., 2015).

Some studies explored the effects of both anodal tDCS and 5 Hz rTMS over M1 to reduce motor deficits in MS (Koch et al., 2008; Cuypers et al., 2013; Meesen et al., 2014; Elzamarany et al., 2016). It has been shown that 5 Hz rTMS over M1 improved hand dexterity in MS patients with cerebellar symptoms (Koch et al., 2008). Intriguingly, the beneficial effect of high frequency rTMS may have raised from enhanced M1 excitatory drive onto the pontine nuclei modulating the cerebro-pontine-cerebellar network (Schwarz and Thier, 1999) and possibly counteracting the reduced cerebellar inputs to these structures due to demyelination. Similarly, another study showed that two consecutive daily sessions of 5 Hz rTMS on M1 improved hand dexterity in a group of RR and progressive MS patients, with a more pronounced effect in RR-MS (Elzamarany et al., 2016). Of the two studies exploring the effects of anodal tDCS, one study evidenced that anodal tDCS could increase cortico-spinal output (Cuypers et al., 2013) and the other study showed that a single concurrent M1 anodal tDCS session had no beneficial effects on a unimanual motor sequence task in a group of RR-MS and progressive MS (Meesen et al., 2014). In addition, repeated 5 Hz rTMS on M1 for 5 consecutive days over 2 weeks may induce beneficial effects also in other dysfunctions, such as lower urinary tract involvement (Centonze et al., 2007b). Notably, other motor symptoms including gait disturbances may respond to NIBS, as five consecutive sessions of high frequency rTMS over the left dorsolateral prefrontal cortex (DLPFC) improved gait in a RR-MS patient (Burhan et al., 2015).

In MS, neuropathic pain and both positive and negative sensory symptoms, including paresthesia and hypoanestesia, are frequently observed and scarcely responsive to pharmacological treatment. It has been reported that 5 consecutive days of anodal tDCS on M1 may improve neuropathic pain in MS (Mori et al., 2010b). In addition, another study reported that 3 consecutive days of anodal tDCS over the DLPFC reduced neuropathic pain in MS (Ayache et al., 2016). Finally, tactile hypoanesthesia improved in RR-MS after 5 consecutive days of anodal tDCS over the somatosensory cortex (Mori et al., 2013).

Fatigue represents another disabling symptom frequently observed in MS patients, occurring in all disease stages (Krupp and Pollina, 1996; Kos et al., 2004; Lerdal et al., 2007). The nature of fatigue in MS is multifactorial and it has been related to different pathophysiological mechanisms, including structural or functional brain alterations (Ayache and Chalah, 2017). Different studies explored the effects of anodal tDCS on fatigue in MS. The available studies differ in terms of parameters and stimulation sites, including M1 (Ferrucci et al., 2014), somatosensory cortex (Tecchio et al., 2014), left DLPFC (Saiote et al., 2014; Chalah et al., 2015, 2017), and right posterior parietal cortex (Chalah et al., 2017). Overall, whereas the results suggest that tDCS may represent a promising approach to treat fatigue, further studies are needed to define the optimal stimulation parameters and site (Lefaucheur et al., 2017).

Finally, only few studies explored the use of NIBS for treating cognitive deficits associated with MS. One study showed that 10 daily sessions of anodal tDCS over the left DLPFC significantly improved concurrent cognitive training (Mattioli et al., 2016). Another study explored the effects of 5 Hz rTMS over the right DLPFC on working memory deficits (Hulst et al., 2016). In that study, cognitive performance improvement was associated with a reduced aberrant hyperactivation of the prefrontal areas observed in MS patients. It is worth noting that although cognitive activities are subserved by diffuse networks, focal modulation of a node may induce functional changes in remote regions (Siebner et al., 2009).

## Conclusions

Improved understanding of features inducing brain disconnection in MS (i.e., demyelination, neurodegeneration, inflammation) and those influencing recovery (i.e., plasticity) may help to characterize the underlying pathophysiology. As neuroinflammation could induce brain connectivity dysfunction and impair network reorganization, contrasting inflammation may hinder connectivity disruption in MS. Furthermore, strategies aimed at promoting plasticity could be particularly relevant in MS, as plasticity reserve is reduced in these patients. Although NIBS could represent a promising approach for treating different symptoms in MS, it will be useful to identify the brain areas that should be stimulated and relate the lesion site to therapeutic response, establish the need to perform consecutive stimulation sessions and better predict the individual response to NIBS, also considering that in MS plasticity is altered in response to inflammation. It is therefore crucial to understand how focal modulation of brain activity by NIBS can enhance network efficiency. In particular, identifying anatomical and functional principles determining how local perturbations affect large-scale neural activity (Sale et al., 2015) may help to predict the impact of NIBS on brain network reorganization (Gollo et al., 2017). This could help to define whether restoring the original networks or promoting alternative circuits could be the best strategy for recovery, and therefore to use NIBS with an increasingly tailored approach.

## Author contributions

MS and EI: work conception and design, drafting the work, work revision, final approval and global agreement; LG: work conception and design, work revision, final approval and global agreement; FB, PM, GM, and DR: work revision, final approval and global agreement; DC: work conception and design, guarantor of integrity of entire study, manuscript revision for important intellectual content, final approval.

### Conflict of interest statement

The authors declare that the research was conducted in the absence of any commercial or financial relationships that could be construed as a potential conflict of interest.
